# Identification and characterization of *ANO9* in stage II and III colorectal carcinoma

**DOI:** 10.18632/oncotarget.4979

**Published:** 2015-07-20

**Authors:** Chunxiang Li, Sanjun Cai, Xishan Wang, Zheng Jiang

**Affiliations:** ^1^ Laboratory of Medical Genetics, Harbin Medical University, Harbin, Heilongjiang, China; ^2^ Department of Colorectal Surgery, Fudan University Shanghai Cancer Center, Shanghai, China; ^3^ Department of Colorectal Cancer Surgery, The 2nd Affiliated Hospital, Harbin Medical University, Harbin, Heilongjiang, China; ^4^ Colorectal Cancer Institute of the Heilongjiang Academy of Medical Sciences, Harbin, Heilongjiang, China

**Keywords:** ANO9, colorectal cancer, prognosis, metastasis

## Abstract

Background and Objectives: The precise role and potential underlying mechanisms of *anoctamin 9* (*ANO9*) remain largely unknown. This study aims to characterize the role and oncogenic mechanisms of *ANO9* in stage II and III colorectal cancer (CRC).

Methods: We examined the expression of *ANO9* in colorectal cancerous tissues and cells using real-time quantitative PCR and immunohistochemistry, respectively. Multiple cellular and molecular approaches such as gene transfection, CCK-8 assay, flow cytometry, and invasion assay were also performed to explore its oncogenic mechanisms. Furthermore, the clinical significance of *ANO9* in clinical CRC specimens was assessed by clinical correlation and survival analyses.

Results: Lower expression of *ANO9* messenger RNA (mRNA) was frequently detected both in CRC tissues with recurrence and metastasis-derived cell lines. Compared with matched nontumorous tissues, lower expression of ANO9 protein was observed in tumors, which was significantly correlated with tumorigenesis (*p* < 0.05). In vitro functional studies showed that *AN*O9 contributed to tumor cell proliferation, apoptosis, and invasion. Moreover, investigation of clinical CRC specimens showed that ANO9 were markedly overexpressed in metastatic tissue compared with primary tissue. Decreased expression of *ANO9* was correlated with poor prognostic outcomes.

Conclusions: This study highlighted the role of *ANO9* in progression and metastasis of stage II and III CRC. These findings suggested that up-regulation of *ANO9*, as a metastasis-related gene, could be a novel approach for inhibiting CRC progression.

## INTRODUCTION

Colorectal cancer (CRC) is the third most prevalent cancer type in the world [1] and fourth leading cause of cancer related deaths [2]. Stage II and III tumors together represent approximately 70% of CRC patients [3]. Although multidisciplinary therapy successfully induce clinical remission in approximately 60% of stage II and III cases, 40-50 % of patients will relapse, and most of them will die from secondary diseases [3, 4]. One third of CRC patients without histological evidence of lymph node involvement die within five years after surgery from distant metastasis or local recurrence [5], suggesting that pathological stage alone may not predict the clinical course of CRC adequately. Therefore, it is necessary to explore the novel molecular markers for identifying aggressive phenotypes within stage II and III CRC.

*ANO9* (*anoctamin 9*), also known as *TMEM16J*, is located in chromosome 11 band p15. It is a member of the *TMEM16* family, which plays key roles in a variety of physiological functions that range from ion transport to phospholipid scrambling and to regulation of other ion channels. Although the first two family members, *ANO1* and *ANO2*, were functionally characterized, which are involved in transepithelial ion transport, olfaction, phototransduction, smooth muscle contraction, nociception, cell proliferation and control of neuronal excitability, until now, the role of *ANO9* remains poorly understood and controversial [6].

The aim of the present study was to investigate the role and oncogenic mechanisms of *ANO9* in human CRC progression. Furthermore, the clinical significance of *ANO9* was also addressed in this study.

## RESULTS

### Differential expression of ANO9 in CRC samples and cell lines

**Figure 1:**
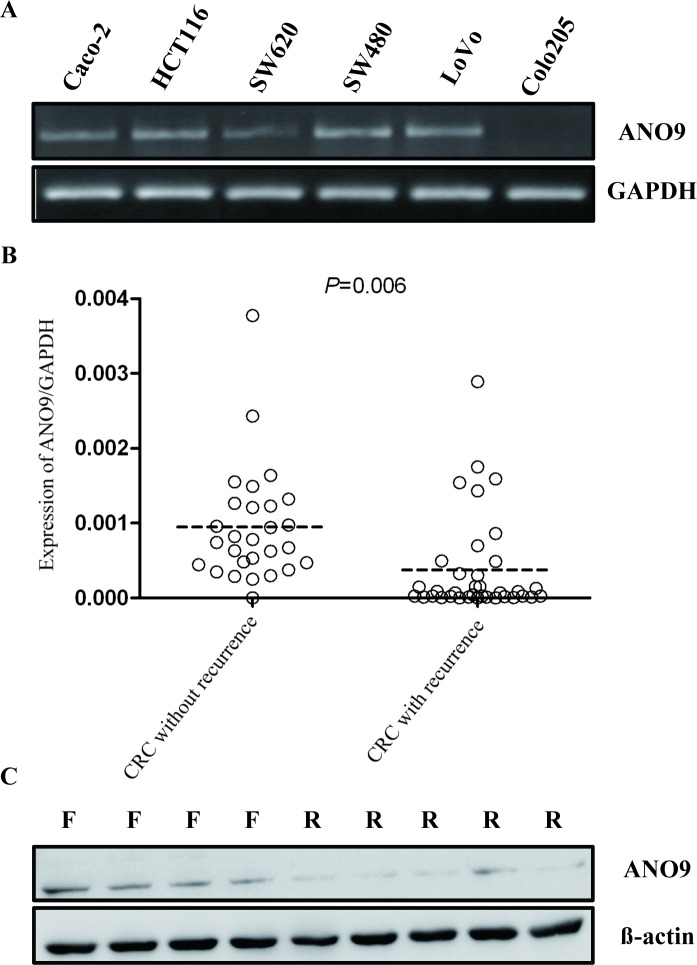
**A.** *ANO9* expression in 6 CRC cell lines was detected by RT-PCR. *GAPDH* was amplified as an internal control. **B.** In recurrent CRC, *ANO9* mRNA levels were significantly decreased compared to the CRC without recurrence (Wilcoxon Signed Ranks Test, *P* < 0.05). **C.** Western blots analysis of ANO9 protein expression in CRC tissues. R indicates recurrence samples; F, samples free of recurrence.

Relative quantities of *ANO9* mRNA in CRC cell lines were expressed as N-fold difference in relation to LoVo and normalized to the *GAPDH* as a reference gene. The *ANO9* mRNA expression in SW480, HCT116, and Caco-2 were increased 2.7-, 1.8- and 1.1-fold, respectively, compared with that of LoVo. Whereas *ANO9* mRNA levels of SW620 and Colo205 were decreased 0.6- and 0.1-fold, respectively. To provide visualized evidence for *ANO9* mRNA expression, RT-PCR was also used and the same results were obtained (Figure 1A). In 64 fresh samples, *ANO9* mRNA expression was significantly lower in recurrent CRC tissues than in those without recurrence (*P* = 0.006; Figure 1B). Moreover, random CRC samples were also selected for Western blotting, and the same results were obtained (Figure 1C).

**Figure 2:**
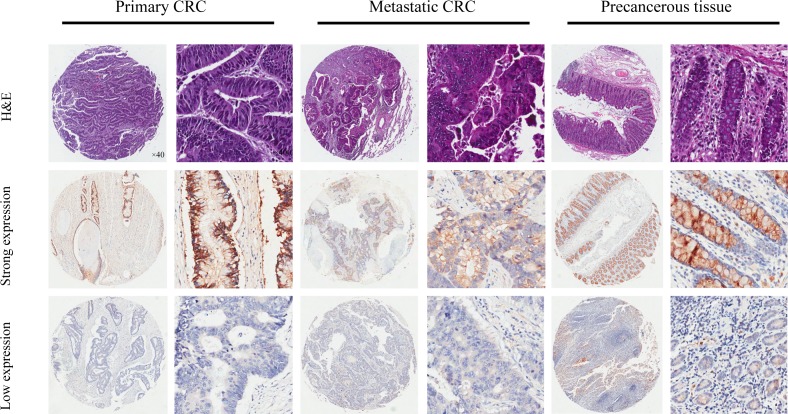
Representatives of ANO9 expression in consecutive TMA slides consisting of 75 non-tumor, primary, and metastatic CRC tissues detected by immunohistochemistry (magnification×40 or 400) In most cases, ANO9 expression was often stronger in the cytoplasm of primary CRC cells compared with metastatic cancer cells. And adjacent non-cancer tissue is detected with stronger ANO9 expression compared with both type of cancer tissues.

By use of a tissue microarray (75 cores) we investigated the protein expression of ANO9 in primary and metastatic cancer specimens and their matched nontumor counterparts. The tumorous or non-tumorous mucosa-specific staining was semi-quantitatively scored by the intensity and the percentage of positive staining. As shown in Figure 2, ANO9 protein expression was detected mainly in cytoplasm of cells. Analysis of ANO9 protein level in the 61 CRC tissues (28 primary tissues and 33 metastatic tissues) revealed that 25% (15/61) of samples demonstrating strong (2+ and 3+) intensities and 75% (46/61) low (- and 1+) intensities. The positive expression of ANO9 in surrounding nontumor tissues was significantly higher than that in tumorous tissue (*P* < 0.001).

### ANO9 decreased cell growth, inhibited cell cycle, and promoted cell apoptosis

**Figure 3:**
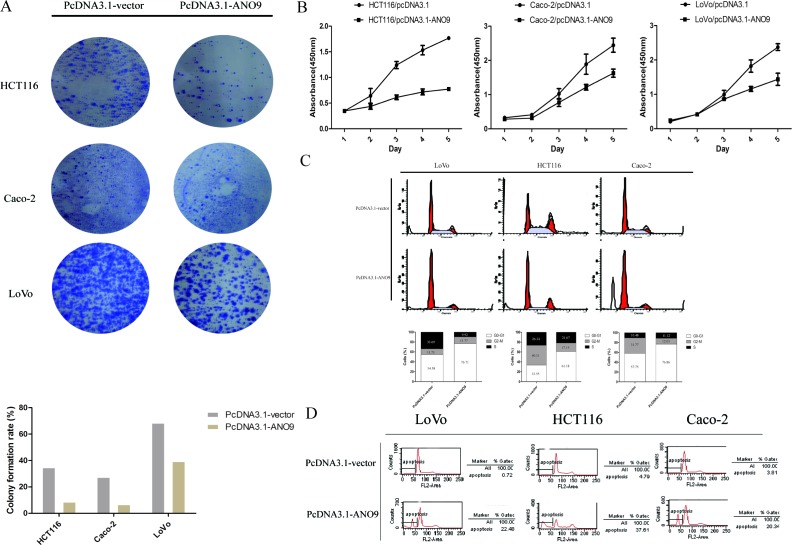
**A.** Less and smaller clones were formed in ANO9-transfected cells by crystal violet staining compared with empty-vector-transfected cells. **B.** CCK-8 assay detected the growth condition of ANO9-transfected cells and empty-vector-transfected cells. Points, means of three separate experiments. Bars, standard deviation (SD) (*P* < 0.05). **C.** *ANO9* over-expression caused a significant accumulation of cells in the G0-G1 phase and a marked decrease in the G2-S phase compared with empty-vector-transfected cells (*P* < 0.05) by flow cytometry. **D.** *ANO9* over-expression promoted cell apoptosis using PI staining to label apoptotic cells.

To characterize the role of *ANO9* in CRC development, the full-length cDNA of the gene was cloned into expressing vector pCDNA3.1 and stably transfected into human CRC cell line HCT116, Caco-2 and LoVo cells. The expression level of ANO9 in transfected cells was determined by Western blotting (Figure S1). While the empty-vector-transfected cells grew into large clones, the *ANO9*-transfected cells grew slowly (Figure 3A). CCK-8 assay demonstrated that *ANO9* inhibited cell proliferation compared with vector-transfected cells (*P* < 0.05) (Figure 3B). Cell cycle analysis showed that *ANO9* over-expression caused a significant accumulation of cells in the G0-G1 phase, with a concomitant decrease of cells in the G2-S phase compared with empty-vector-transfected cells (*P* < 0.05) (Figure 3C). Moreover, it also revealed that *ANO9* over-expression promoted cell apoptosis using PI staining to label apoptotic cells in 3 CRC cell lines (Figure 3D).

### ANO9 decreased cell invasion

**Figure 4:**
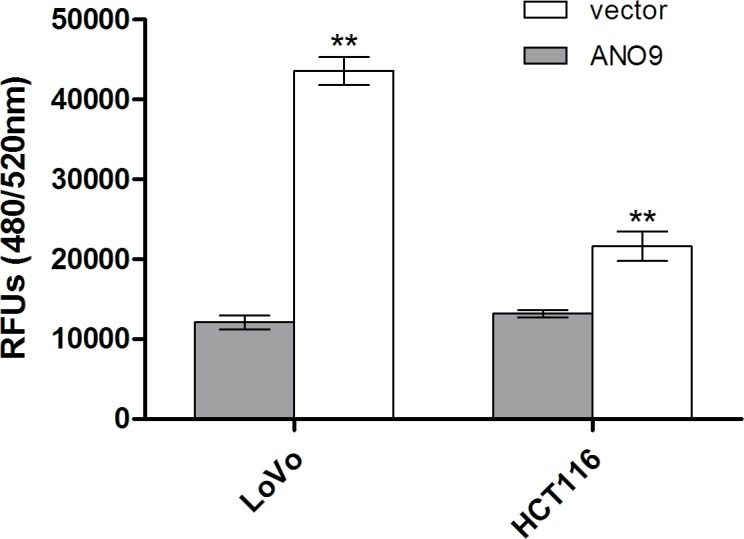
Cell invasion was measured using a QCMTM 24-well cell invasion kit Each experiment was repeated three times, and data represent the mean±SD of three determinations.

Matrigel-transwell assay was used to determine the effect of *ANO9* on cell invasion. PcDNA3.1-vector cells showed about 3.61±0.37-fold and 1.64±0.19-fold penetration rate through the matrigel-coated membrane compared with PcDNA3.1-ANO9 in LoVo and HCT116 cells, respectively (Figure 4), which indicated that *ANO9* reduced the invasion ability of tumor cells.

To confirm the above results, we also tested the effect of *ANO9* knockdown in a CRC cell line, SW480. We first confirmed the knockdown effect on the expression of ANO9 in this cell line (see Supplementary Material, Figure S2A). As expected, *ANO9* knockdown increased cell growth, colony formation, and cell invasion in SW480 cells (see Supplementary Material, Figure S2).

### Correlation between ANO9 expression and clinicopathological features

*ANO9* mRNA expression was significantly associated with cancer recurrence and TNM stage (Table 1). Table 2 showed the relationships between clinicopathological features and ANO9 protein expression in samples. No significant age, sex, size, lymphvascular and perineural invasion differences were observed. However, ANO9 expression was significantly associated with carcinogenesis (*P* < 0.001).

**Table 1:** Relationships between clinicopathological features and *ANO9* mRNA expression in colorectal cancer

Variables	N	ANO9 expression		*P*^a^ value
		High (n=37)	Low (n=27)	
Age				0.223
<80	57	31	26	
≥80	7	6	1	
Sex				1
Male	40	23	17	
Female	24	14	10	
Size				0.314
≤5cm	35	18	17	
>5cm	29	19	10	
Grading				0.460
Well	15	9	6	
Moderate	39	24	15	
Poor	10	4	6	
Type				0.233
Non-mucin-producing cancer	50	31	19	
Mucin-producing cancer	14	6	8	
Location of tumor				0.429
Colon	22	11	11	
Rectum	42	26	16	
Recurrence				<0.001*
Yes	34	9	27	
No	30	28	0	
TNM stage				0.010*
II	27	21	6	
III	37	16	21	
Lymphovascular invasion				0.576
Yes	17	11	6	
No	47	26	21	
Perineural invasion				0.072
Yes	14	5	9	
No	50	32	18	

a *P* value are obtained from χ^2^ test

* Statistically significant, *P*<0.05

**Table 2:** Relationships between clinicopathological features and ANO9 protein expression in colorectal cancer

Variables	N	ANO9 expression		*P*^a^ value
		Strong	Low	
Sex				1
Male	21	3	18	
Female	7	1	6	
Age				0.133
≥65	6	2	4	
<65	22	2	20	
Location				0.527
Rectum	11	1	10	
Colon	17	3	14	
Histologic type				0.549
Non-mucin-producing cancer	26	4	22	
Mucin-producing cancer	2	0	2	
Histologic grading				0.212
Low,moderate-grade	21	2	19	
High-grade	7	2	5	
Tumor size				0.687
>5 cm	5	1	4	
≤5 cm	23	3	20	
Lymphovascular invasion				0.318
Yes	3	1	2	
No	25	3	22	
Perineural invasion				0.678
Yes	1	0	1	
No	27	4	23	
TNM stage				0.212
II	7	2	5	
III	21	2	19	
Type of Cancer				0.085
Primary	28	4	24	
Metastatic	33	11	22	
Type of tissues				<0.001*
Non-tumorous tissue	14	12	2	
Tumorous tissue	61	15	46	

a *P* value are obtained from χ^2^ test

* Statistically significant, *P*<0.05

**Figure 5:**
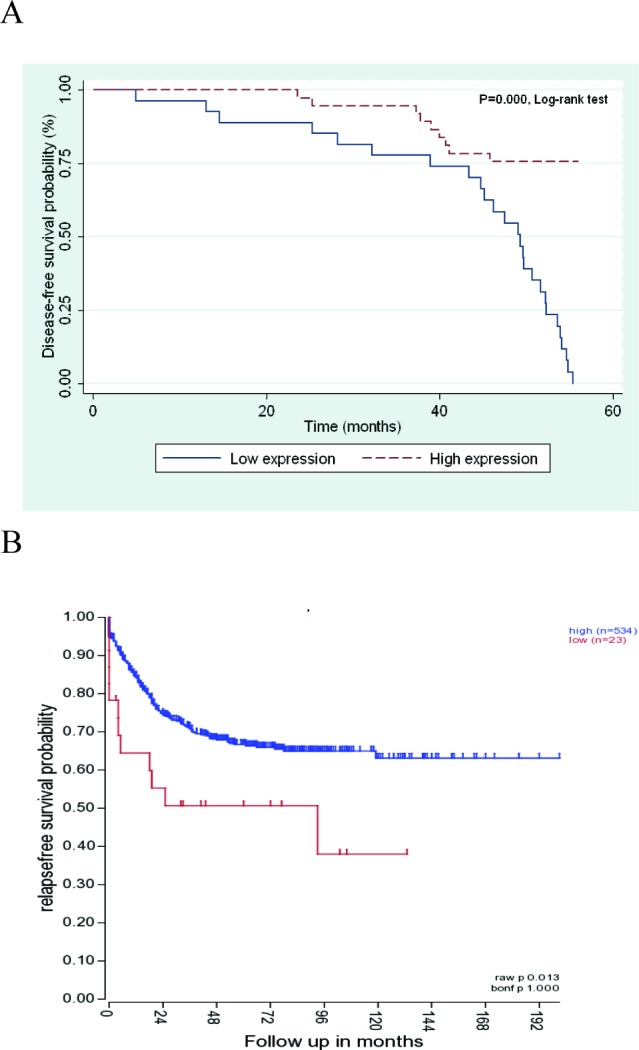
**A.** Patients with low *ANO9* expression (*n* = 37) showed a significantly poorer prognisis than those with high *ANO9* expression (*n* = 27; *P* < 0.05; log-rank test). **B.** Patients with low *ANO9* expression (*n* = 534) showed a significantly poorer prognosis than those with strong expression (*n* = 23) based on R2 applications.

Univariate analysis by Kaplan-Meier plots revealed that high *ANO9* expression was significantly associated with favorable disease-free survival results (*P* < 0.001). Kaplan-Meier curves demonstrate survival distributions were significantly different in CRC regarding to perineural or lymphovascular invasion and TNM stage (*P* < 0.05). Characteristic plots of *ANO9* expression is shown in Figure 5A. Moreover, we also analyzed mRNA expression data of genes in a dataset of 566 samples using the R2 bioinformatic platform (http://r2.amc.nl). It is possible to view the differential expression of genes in a user-defined panel of dataset. We selected the GEO ID: GSE39582 dataset, which aimed to build up a robust molecular classification of mRNA expression profiles (Affymetrix U133Plus2) of a large series of 443 colon cancer. Kaplan-Meier plots revealed that overexpression of *ANO9* expression was significantly associated with favorable relapse-free survival results (*P* = 0.013, Figure 5B). Multivariate Cox analysis indicated that *ANO9* expression, TNM stage, and lymphovascular invasion were independent variables for prognosis of stage II and III CRC patients (*P* = 0.007, 0.001, and 0.003) (Table 3).

**Table 3:** Uni- and multivariate analysis of survival in CRC

Factors	HR (95% CI)	*P* value*
Univariate analysis		
Sex	0.793(0.388-1.620)	0.524
Age	0.167(0.023-1.219)	0.078
Tumor location	1.021(0.514-2.027)	0.954
Tumor size	0.515(0.255-1.036)	0.063
TNM stage	7.479(3.063-18.266)	0.000*
Histologic type	0.635(0.297-1.356)	0.241
Lymphovascular invasion	2.805(1.386-5.677)	0.004*
Perineural invasion	4.374(2.175-8.795)	0.000*
ANO9 expression	0.131(0.058-0.292)	0.000*
Multivariate analysis		
ANO9 expression	0.097(0.033-0.292)	0.001*
TNM stage	9.380(2.857-30.794)	0.003*
Lymphovascular invasion	12.366(3.815-40.077)	0.007*

HR, hazard ratio; CI, confidence interval

* Statistically significant, *P*<0.05

## DISCUSSION

Our study characterizes the role of *ANO9* in human CRC. The *ANO9* expression in recurrent CRC tissues was found significantly lower than in those without recurrence. And the proportion of strong ANO9 protein expressers in non-tumorous tissue was significantly higher than that in tumorous tissue (*P* < 0.05). Furthermore, we investigated the oncogenic mechanisms of *ANO9* in human CRC cell lines, and found that it promotes cell proliferation and tumor invasion *in vitro*. Moreover, survival analysis demonstrated that *ANO9* expression was associated with disease/relapse-free survival time, which confirmed its role in progression of CRC. Multivariate survival analysis showed that strong *ANO9* expression was an independent protection factor.

Although the role of several TMEM16 family members is relatively clear, *ANO9* remain poorly understood. Overexpression of *ANO9* was involved in Ca^2+^-dependent lipid scrambling [11]. On the other hand, it was shown that *ANO9* mediates Ca^2+^-dependent currents [12]. Schreiber *et al.* found that *ANO9* reduced both basal and ATP-induced anion conductance, and inhibited the activity of *ANO1* [13]. As known to us, *ANO1* mediates cell proliferation, signal transduction and tumorigenesis [14-22]. Especially, it induces *MAPK* and contributes directly to cancer progression [15], whereas *ANO9* could inhibit the activity of *ANO1* and other anoctamins [13], which implied that *ANO9* might inhibit malignant progression. In this study, we found that low expressers of *ANO9* exhibited a higher recurrence trend, with a nearly 10-fold higher risk of metastases (HR,10.31; *P* < 0.05). Although the exact function of *ANO9* is still unclear, on the basis of present work, *ANO9* plays a tumor-suppressor role in stage II and III CRC by inhibiting cell cycle, reducing proliferation, promoting apoptosis and decreasing cancer cell invasion.

In view of the comprehensive data presented in this study, we propose that low-expression of *ANO9* indicates poor prognosis in stage II and II CRC patients (Figure 1), which contributes to identifying subsets of patients with aggressive tumors. The role of adjuvant chemotherapy in those patients with stage II tumors is still unclear [23, 24]. To maximize the benefits of adjuvant therapy, an independent prognostic marker could be helpful in identifying aggressive phenotypes within stage II CRC. To our knowledge, it is the first time to explore the role of *ANO9* in tumors, which might help to identify a subset of patients at high-risk of recurrence and these patients may therefore benefit from more aggressive treatment.

Taken together, in this study we showed that reduction of *ANO9* has an important role in the tumorigenesis and cancer progression of stage II and III CRC. Better understanding of the pathophysiological functions of *ANO9* may lead to a much more effective management of CRC via precise prognostication and molecularly targeted treatment.

## MATERIALS AND METHODS

### CRC samples and cell lines

From April 2000 to November 2004, 64 fresh CRC tissues, 36 with recurrence and 28 without recurrence, were collected immediately after operation at the Fudan University Shanghai Cancer Center (Shanghai, China). For immunohistochemical analysis, we selected 28 patients with CRC who underwent surgery at hospitals that cooperated with Shanghai Outdo Biotech Co., Ltd. during 2005-2007. Clinicopathaological characteristics of the research subjects are shown in Table 1 and 2. Patients were only included in this study if they had provided written consent to participate in the study after receiving written information regarding its course and purpose. Approval for the study was received from the Ethics Committee of the host institution.

Human CRC cell lines SW480, SW620, LoVo, Colo205, HCT116 and Caco-2 were obtained from American Type Culture collection (ATCC, Manassas, VA). The method of cell culture was described previously [7].

### mRNA analysis by qPCR

Total RNA was isolated with an RNeasy Mini Kit (Qiagen GmbH, Hilden, Germany) and treated with Dnase. According to the manufacturer's instructions, cDNAs were synthesized with Oligo-dT primers (Promega, Madison, WI). The ANO9-specific primers used were 5′-AGGACTTCCAGGACCCTGAT-3′ (forword) and 5′-CACGTGCTCAAAGAGGATGA-3′ (reverse). Glyceraldehyde-3-phosphate dehydrogenase (GAPDH) served as a control for normalization of gene expression and was amplified using primers 5′-GAAGGTGAAGGTCGGAGTC-3′ (forward) and 5′-GAAGATGGTGATGGGATTTC-3′ (reverse). The method of qPCR was described previously [8].

### Western blotting

The cells were lysed in lysis buffer (PBS containing 1% Triton X-100, protease inhibitor cocktail, and 1 mmol/L phenylmethylsulfonylfluoride) at 4°C for 30 minutes. Equal quantities of protein were subjected to SDS-PAGE. After transfer to Immobilon-P transfer membrane, successive incubations with anti-ANO9 and anti-β-actin, and horseradish peroxidase-conjugated secondary antibody were carried out. The immunoreactive proteins were then detected using the ECL system. Bands were scanned using a densitometer (GS-700; Bio-Rad).

### Tissue microarray (TMAs) construction and Immunohistochemistry

The colonic tissue microarray was constructed by Shanghai Biochip Co, Ltd, as described previously [9]. Immunohistochemical staining was performed using two-step method. The sections were deparaffinized and rehydrated. Antigen retrieval was performed by autoclaving the slides in 10 mM citric acid buffer. A polyclonal rabbit antihuman ANO9 antibody (dilution 1:500) was obtained from Abcam (Cambridge, UK).

Cytoplasm staining was measured for this antibody. Positive cells were counted by 2 pathologists who were blind to clinical outcome. For clinicopathological correlation, we used a 4-tiered scoring system (negative to 3+), which took into account the percentage of positive cells and staining intensity as described previously [7]. The detailed approach was used to generate a score for each tissue core as follows: no staining or staining < 10% of tumour cells (score 0), faint/barely perceptible partial staining > 10% of tumour cells (score 1+), weak-to-moderate staining > 10% of tumour cells (score 2+), and strong staining > 10% of tumour cells (score 3+). We separately interpreted ANO9 - and 1+ as ‘low expression’ and 2+ and 3+ as ‘strong expression’.

### Plasmid constructs and transfection

ANO9 was polymerase chain reaction (PCR) amplified and cloned into expression vector pcDNA3.1(+) (Invitrogen, Carlsbad, CA) and stable ANO9-expressing clones in CRC cells were selected for further experiments.

### Colony forming assay

For clonogenic assay, 1×10^3^ cells were seeded into 6-well tissue culture plates and left to form clones over a period. Cultures were stained with 0.1 % crystal violet, and the number of clones in a 2×2 cm grid (on the culture plates) was scored to determine the clone-forming ability of the cells. Clones containing over 50 cells were counted. Each result was performed in triplicate.

### Cell growth assay

Cell growth was analyzed using a WST-8 Cell Counting Kit-8 (Dojindo, Kumamoto, Japan). Cells (2×10^3^) suspended in RPMI1640 medium (100μl) containing 10% fetal bovine serum were seeded in 96-well plates and incubated. CCK-8 solution (10μl) was added to each well and the cultures were incubated at 37°C for 90 min. Absorbance at 450 nm was measured using an immunoreader. The results were plotted as means ± SD of three separate experiments having five determinations per experiment for each experimental condition.

### Cell cycle analysis

Cell cycle distribution was analyzed by flow cytometry. Cells were trypsinized, rinsed with PBS, fixed with 70% ethanol at 4°C overnight, and treated with RNaseA (0.02 mg/ml) in the dark at room temperature for 30 min. Cells were resuspended in 0.05 mg/ml propidium iodide and analyzed with flow cytometry (Becton Dickinson). The results were analyzed using software (FCSExpress 4 Flow Research Edition) to determine the distribution of different cell cycle phases.

### Apoptosis assay by flow cytometry

Cells were collected and washed with ice cold PBS twice, and resuspended at a density of 1×10^6^ cells/mL in ice cold PBS. One milliliter was transferred into a new tube and 5 μL of 50 μg/mL PI staining solution was added. The tubes were gently mixed and incubated for 15 min at RT in dark conditions. The samples were then analyzed using flow cytometry within 1 h. Unstained cells were used as negative control.

### Cell invasion assay

The cell invasion assay was performed with QCM 24-well Invasion Assay kit (Chemicon International). This cell invasion assay was performed in an invasion chamber, based on the Boyden chamber principle. This kit contains 24 inserts and each insert contains an 8-μm pore size polycarbonate membrane coated with a thin layer of ECM. The method was described previously [10].

### RNAi

Oligonucleotides containing siRNA sequences of the target genes were designed, and named siANO9-1, siANO9-2 and sicontrol (see Supplementary Material, Table S1). The annealed oligos were cloned into the pSUPER.retro vector (Oligoengine, Boston, MA, USA) and sequenced. The recombinant pSUPER-sh-RNA vectors were transfected into CRC cells with LipofectamineTM 2000 transfection reagent (Invitrogen). Stable clones were selected with 1.0 μg/ml puromycin (Sigma-Aldrich) for 7 days.

### Statistical analysis

Statistical analyses were conducted with Stata (version SE/10; StataCorp, College Station, TX). The association among categorical data was analyzed by using the χ^2^ test. Survival curves were generated by the Kaplan-Meier method, and univariate survival distributions were compared with the use of the log-rank test. The multivariate Cox proportional hazards model was used for detection of independent prognosticator. The 2-tailed P value for significance was established at 0.05.

## SUPPLEMENTARY MATERIAL FIGURES AND TABLE


