# Supplementary material for: Identification and characterization of ANO9 in stage II and III colorectal carcinoma
Source: Oncotarget. 2015 Jul 20;6(30):29324–34. doi: 10.18632/oncotarget.4979 (PMC4745729; doi:10.18632/oncotarget.4979)
Supplement: Supplementary file 1 [file oncotarget-06-29324-s001.pdf]

Identification and characterization of *ANO9* in stage II and III colorectal carcinoma

Supplementary Material

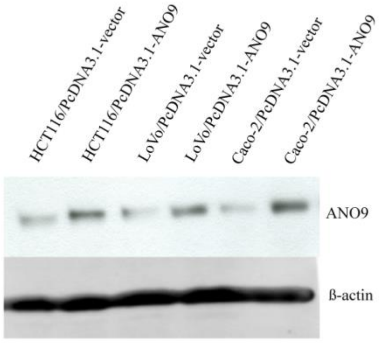

Figure S1: Western blot was used to detect ANO9 expression in ANO9-transfected CRC cell lines and empty vector-transfected cells. β-actin were used as loading controls.

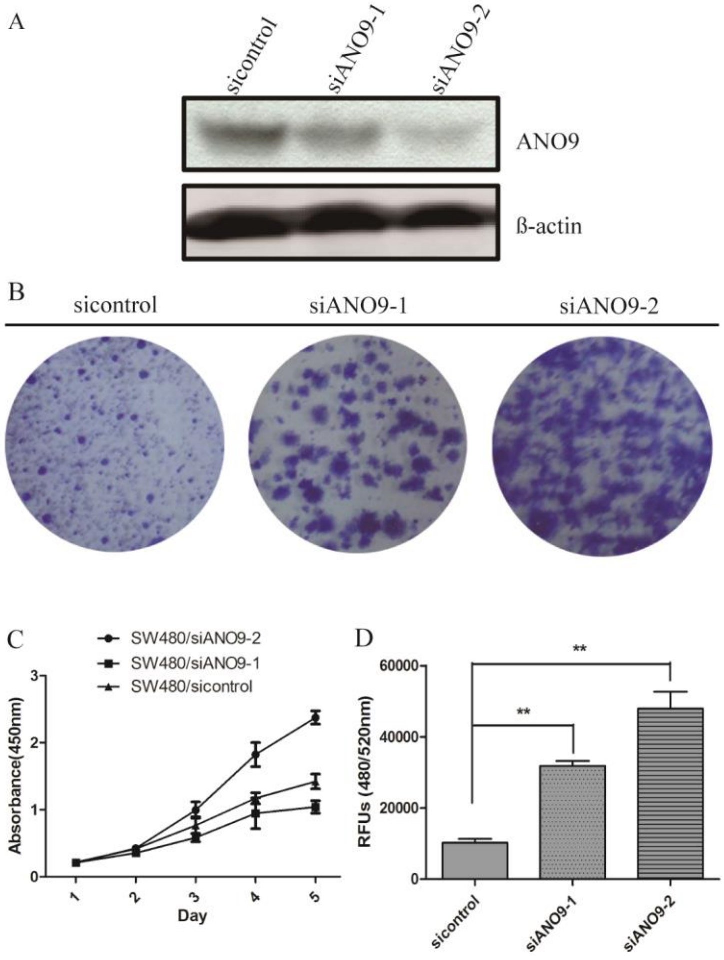

Figure S2: ***A.*** Knock-down efficiency was evaluated by Western blot. ***B.*** More and larger clones were formed in *ANO9* knock-down cells. ***C.*** CCK-8 assay detected the growth condition of *ANO9* knock-down cells. Points, means of three separate experiments. Bars, SD ($P < 0.05$). ***D.*** *ANO9* knock-down cells showed increased invasion ability.

A

Breakdown by Health State

		Hs.501622
adrenal tumor	0	0/12655
bladder carcinoma	0	0/17584
breast (mammary gland) tumor	21	2/93090
cervical tumor	0	0/34484
chondrosarcoma	0	0/82838
colorectal tumor	62	7/112517
esophageal tumor	115	2/17245
gastrointestinal tumor	42	5/118498
germ cell tumor	0	0/263230
glioma	0	0/107194
head and neck tumor	14	2/133826
kidney tumor	0	0/68872
leukemia	21	2/94479
liver tumor	0	0/96023
lung tumor	29	3/102765
lymphoma	27	2/72196
non-neoplasia	31	3/96623
normal	13	46/3328811
ovarian tumor	91	7/76185
pancreatic tumor	0	0/105004
primitive neuroectodermal tumor...	0	0/127001
prostate cancer	0	0/103844
retinoblastoma	0	0/46439
skin tumor	0	0/125373
soft tissue/muscle tissue tumor	0	0/125265
uterine tumor	33	3/90107

B

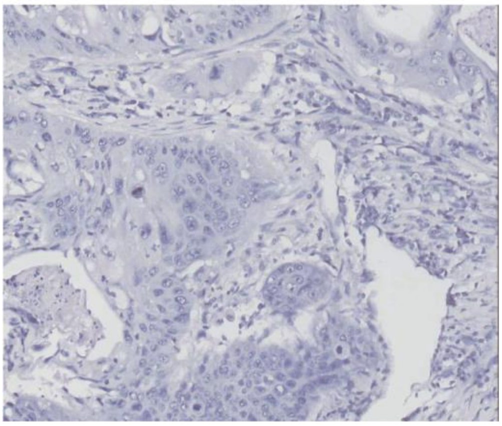

Figure S3: Evaluation of the specificity of the antibody. ***A.*** ANO9 is not expressed in pancreatic carcinoma based on NCBI database/UniGene. ***B.*** No ANO9 protein expression in pancreatic tumor tissue.

Table S1. siRNA sequence

Name	siRNA sequence
si-control	CTGGCATCGGTGTGGATGA
si- ANO9-1	AACTCTACTGTCGACTGCCTC
si- ANO9-2	AACTTCTCCGAGCAGTTCTGG
